# Ten-year pathology-based cancer registry at a tertiary referral hospital in Kenya (2015–2024): distribution of cancers and completeness of pathology reporting

**DOI:** 10.1186/s12885-026-15813-w

**Published:** 2026-03-09

**Authors:** Brian O. Ayara, Samuel G. Mukono, Rodney D. Adam, Shahin Sayed

**Affiliations:** Department of Pathology, Aga Khan University Hospital, P.O. Box 30270-00100, Nairobi, Kenya

**Keywords:** Cancer registry, Kenya, Cancer epidemiology, Pathology-based registry, Histopathology, Tumor distribution, Cancer prevalence, Aga Khan University

## Abstract

**Background:**

Population-based cancer registries remain limited in low- and middle-income countries (LMICs). In Kenya, their coverage is below the 20% threshold recommended by the International Agency for Research on Cancer. Pathology-based registries can provide complementary data in such contexts. This study describes the distribution of cancers diagnosed at a tertiary referral hospital in Nairobi over ten years, and evaluates the completeness of pathology reporting for selected cancers using College of American Pathologists (CAP) protocols.

**Methods:**

We conducted a retrospective review of all histologically confirmed cancers at the Aga Khan University Hospital, Nairobi, from 2015 to 2024. Data were extracted from the institutional database. The data included region of origin, demographics, cancer site, and histologic diagnosis. Completeness of reporting was assessed in stratified random samples of breast and colorectal cancer reports against the College of American Pathologists (CAP) cancer reporting protocols.

**Results:**

A total of 32,445 cancer cases were diagnosed over the study period, including 18,899 females and 13,546 males (58.3% and 41.7%, respectively). The median age at diagnosis was 52 years (Interquartile range 41–64) in females and 61 years (Interquartile range 47–71) in males, with an overall median age of 56 years (Interquartile range 43–68). Most cases occurred in the decades between 51 and 60 years (6816/32,445) and 61–70 years (6556/32,445). Overall, breast cancer was the most frequent malignancy (7236/32,445; 22.3%), followed by esophageal (3839/32,445; 11.8%) and prostate cancer (3023/32,445; 9.3%). Among women, breast (6926/18,899; 36.6%), cervical (2230/18,899; 11.8%), and esophageal cancers (1610/18,899; 8.5%) predominated, while among men, prostate (3023/13,546; 22.3%), esophageal (2229/13,546; 16.5%), and non-Hodgkin lymphoma (957/13,546; 7.1%) were most common. Pediatric cancers (≤ 19 years) accounted for 1325 cases (4.1%). Completeness of pathology reporting exceeded 95% for most required elements, and it improved from 83.3% in 2015 to over 98% in 2024.

**Conclusion:**

This large pathology-based dataset highlights the cancer burden in Kenya, with breast and prostate cancers predominating among females and males, respectively. There are regional disparities in sources of referral for certain cancer types. Pediatric malignancies represented a small proportion of cases. Pathology reporting for key data elements was generally of high completeness.

**Supplementary Information:**

The online version contains supplementary material available at 10.1186/s12885-026-15813-w.

## Introduction

Cancer incidence continues to rise globally and particularly in Africa. According to the GLOBOCAN 2022 report, there were an estimated 20 million new cases and 9.7 million deaths worldwide in 2022, with a disproportionate share of mortality occurring in low- and middle-income countries [1]. In Kenya, 44,726 new cases and 29,317 deaths were recorded in 2022 against a population of 56 million, underscoring the growing burden of the disease (Bray et al.,). In response, the Kenya National Cancer Control Strategic Plan (2023–2027) identified “Strategic Information, Registration, Surveillance and Research” as one of its five pillars, recognizing the central role of reliable data in guiding cancer control strategies [2].

Cancer registries remain the cornerstone of such efforts, with population-based registries considered the gold standard for generating high-quality cancer incidence and survival data [3]. They systematically collect all cases within defined catchment areas and provide critical information for public health planning. However, establishing and maintaining population-based registries is resource intensive, and coverage in sub-Saharan Africa remains limited [4]. Kenya currently has only two high-quality population-based registries—Nairobi and Eldoret—covering approximately 11% of the population, well below the 20% threshold recommended by the International Agency for Research on Cancer [2]. Hospital- and pathology-based registries, although more limited in scope, provide complementary information in contexts where population-based registries are lacking [5]. Pathology-based registries, in particular, capture histologically confirmed cancers and offer reliable diagnostic information. Studies from Pakistan and other LMICs have shown that trends observed in large pathology-based datasets often parallel those reported by population-based registries, supporting their utility in cancer surveillance [6]. Despite inherent biases, such data can highlight distribution patterns, inform clinical decision-making, and identify capacity gaps.

In Kenya, histopathology services are available in a limited number of public and private tertiary facilities, and many public and private hospitals without in-house capacity refer specimens to tertiary laboratories, including private-sector referral laboratories like the Aga Khan hospital laboratory. A diagnostic pathology survey in 2018 reported that 45% of public hospitals lacked capacity for histopathology testing, leading to significant reliance on private sector laboratories for cancer diagnosis [7]. Consequently, pathology-based registries within tertiary referral centers provide an opportunity to capture diverse cases from both public and private facilities nationwide. Aga Khan University Hospital, Nairobi (AKUH) is a private tertiary referral hospital, and patients receiving direct clinical care are more likely to include middle- and higher-income groups. However, because its pathology laboratory serves as a regional referral center it receives many specimens from external public hospitals serving lower-income populations, where histopathology services are limited. With additional coverage through social health insurance schemes, the pathology-based registry therefore reflects a mixed referral population rather than exclusively AKUH clinical patients.

The completeness of pathology reports is particularly critical. Missing information on margins, grade, or biomarker testing can compromise staging accuracy and treatment planning, and multidisciplinary tumor boards rely on these data to guide evidence-based care [8, 9]. Evaluating the consistency of reporting offers insight into the quality of cancer diagnostics and opportunities for improvement. This study therefore aimed to describe the distribution of cancers diagnosed at the AKUH Nairobi, from 2015 to 2024, and to assess the completeness of pathology reporting for selected cancers using the College of American Pathology cancer reporting protocols. By leveraging a large pathology-based dataset, this work contributes baseline evidence to complement population-based registries and inform national cancer control planning in Kenya.

## Methods

### Study design and setting

We conducted a descriptive retrospective study over a ten-year period of all cancer cases diagnosed from January 2015 through December 2024 in the pathology department of the Aga Khan University Hospital, Nairobi (AKUHN). The AKUHN is a Joint Commission International (JCI) accredited tertiary referral hospital with capacity for histopathology, hematopathology, immunohistochemistry, and molecular diagnostics. The laboratory processes 3000–4000 cancer cases annually and receives specimens from 158 institutions nationwide, including private, public, and faith-based facilities. All data analyzed in this study were derived exclusively from the Aga Khan University Hospital Nairobi pathology department database. Specimens were received both from AKUHN clinical services and from external referring facilities across Kenya, including public, private, and faith-based institutions. No data were extracted from national or population-based cancer registries. The term ‘pathology-based registry’ in this study refers to an institutional database of histologically confirmed cancers maintained within the pathology department.

### Study population

All cases with histologically confirmed cancer diagnosed at AKUHN during the study period were eligible. Exclusion criteria included inconclusive or preliminary diagnoses pending further testing.

### Sampling strategy

For overall cancer distribution, we analyzed all eligible cases (census approach). For assessment of report completeness, we applied stratified random sampling of two cancer types—breast and colorectal. Sample size was calculated using Fisher’s formula, yielding a minimum of 385 reports per stratum at 95% confidence level and 5% margin of error. Random numbers were used to select cases within strata.

### Data collection

Records were retrieved from the hospital electronic databases by trained staff. Extracted data included unique identifiers, year of diagnosis, age, sex, cancer site, and pathology findings. Data were entered into Microsoft Excel for secure management. Completeness of pathology reports was evaluated against CAP cancer reporting protocols, focusing on key diagnostic and prognostic indicators.

### Data quality assurance

Quality control included double-checking entries against source documents, and regular audits. Automated validation ensured completeness of mandatory fields, detection of duplicate entries, and plausibility of date ranges. Incomplete or inconsistent records were flagged and resolved.

### Statistical analysis

Data were analyzed using R version 4.3.0. Categorical variables were summarized as proportions and continuous variables as means or medians. Relative frequencies of cancers were stratified by age, sex, and source of referral. Completeness of pathology reports was expressed as proportions of elements in reports meeting CAP standards.

### Ethical considerations

This study was approved by the Aga Khan University Institutional Scientific and Ethics Review Committee (Ref: 2024/ISERC-125) and licensed by the National Commission for Science, Technology, and Innovation (NACOSTI/P/25/4181523). Informed consent was waived due to the retrospective use of de-identified data. Patient confidentiality was strictly maintained through anonymization and secure data handling.

## Results

### Distribution of cases

A total of 32,445 cancer cases were diagnosed over the 10-year period, comprising 18,899 females (58.3%) and 13,546 males (41.7%). Of the 32,445 cancer cases, about 16,213 (50.0%) were external pathology referrals from both public and private facilities outside Aga Khan University Hospital. The median age at diagnosis was 52 years (IQR 41–64) in females and 61 years (IQR 47–71) in males, with an overall median age of 56 years (IQR 43–68). The highest proportion of cases occurred between 51 and 60 years (6816/32,445) and 61–70 years (6556/32,445; Table 1).

**Table 1:** Cancer cases by age band and sex

Age, years	Female (*n* = 18899)	Male (*n* = 13546)	Overall (*n* = 32445)
Median (IQR)	52 (41–64)	61 (47–71)	56 (43–68)
**Age Range**			
1–10	168 (0.9)	296 (2.2)	464 (1.4)
11–20	393 (2.1)	468 (3.5)	861 (2.7)
21–30	1030 (5.5)	550 (4.1)	1580 (4.9)
31–40	2917 (15.4)	1062 (7.8)	3979 (12.3)
41–50	4382 (23.2)	1731 (12.8)	6113 (18.8)
51–60	4178 (22.1)	2638 (19.5)	6816 (21.0)
61–70	3291 (17.4)	3265 (24.1)	6556 (20.2)
Above 70	2540 (13.4)	3536 (26.1)	6076 (18.7)
Percentage (%)	58.3	41.7	

### Cancer distribution by site and sex

**Fig. 1:**
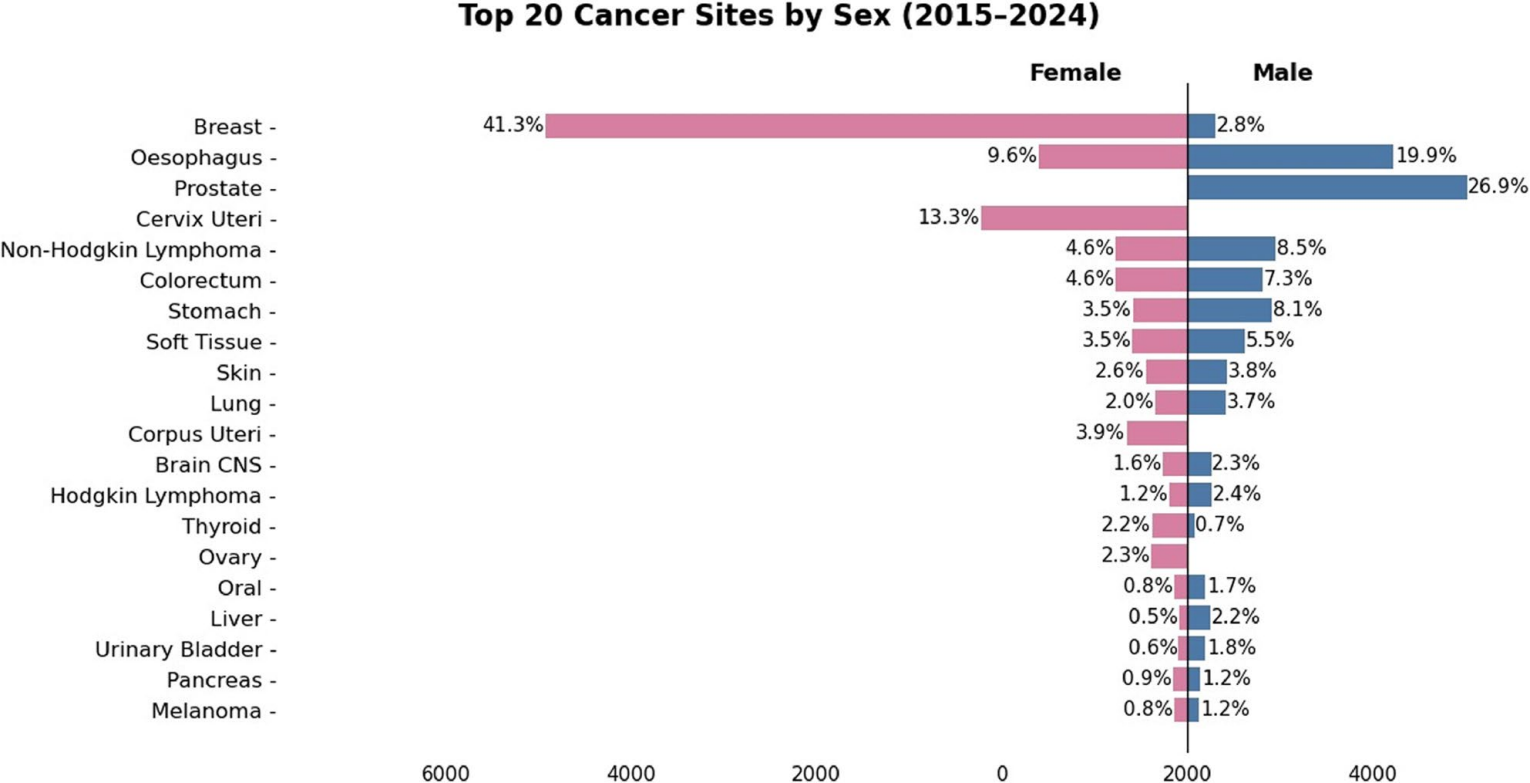
Graphical representation of the distribution of Top 20 Cancer Sites by Sex

**Fig. 2:**
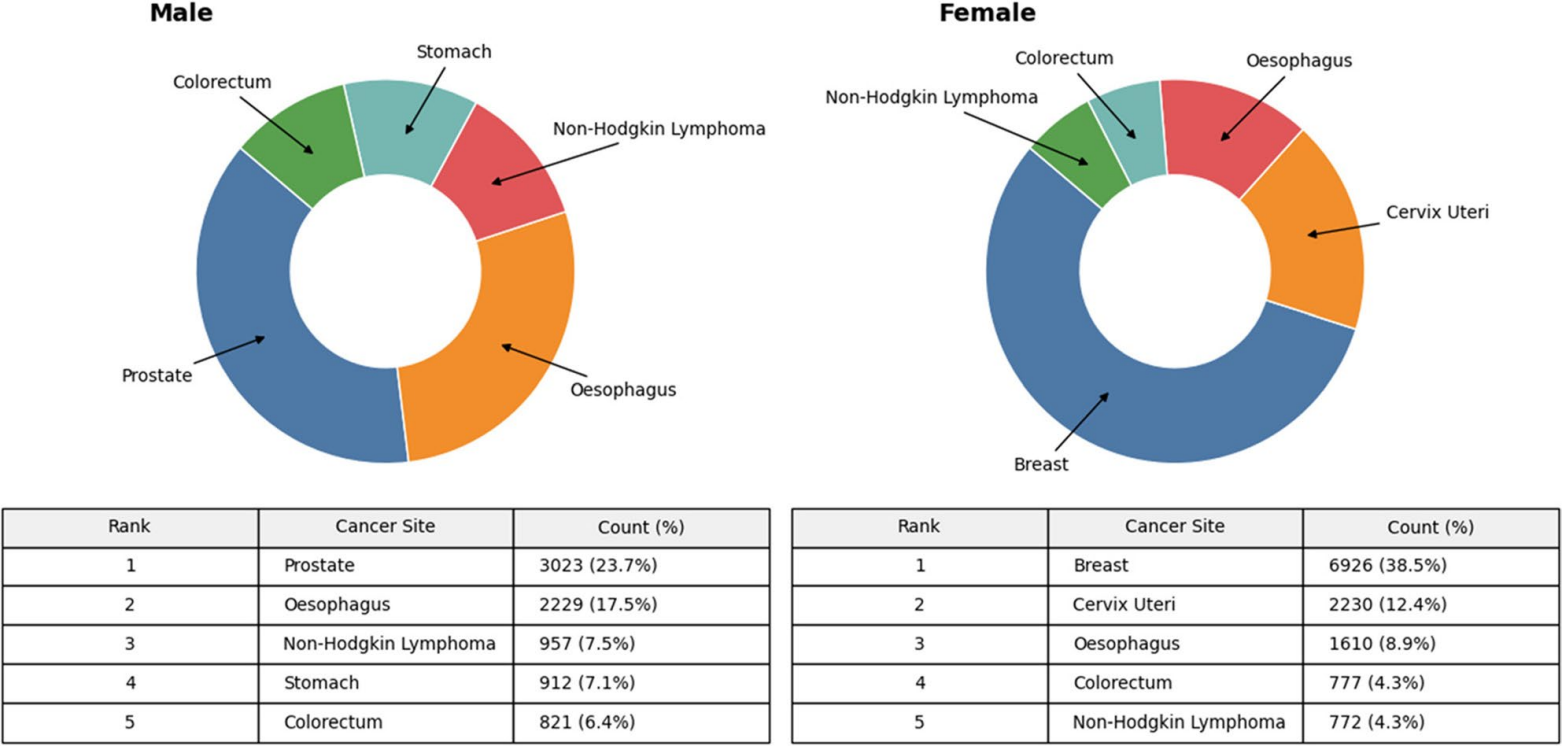
Top 5 common sites of cancer diagnosed in females and males during the study period

The most common malignancy overall was breast cancer (7236/32445; 22.3%), followed by esophageal cancer (3839/32,445; 11.8%) and prostate cancer (3023/32,445; 9.3%). Among females, breast cancer (6926/18,899; 36.6%), cervical cancer (2230/18,899; 11.8%), and esophageal cancer (1610/18,899; 8.5%) were the most frequently diagnosed malignancies. In males, prostate cancer (3023/13,546; 22.3%), esophageal cancer (2229/13,546; 16.5%), and non-Hodgkin lymphoma (957/13,546; 7.1%) predominated (Table 2; Figs. 1 and 2).

**Table 2:** Top 20 cancer cases diagnosed during the period 2015 to 2024

Rank	Cancer Site	Female *n* (%)	Male *n* (%)	*N*
1	Breast	6926 (95.7)	310 (4.3)	7236
2	Esophagus	1610 (41.9)	2229 (58.0)	3839
3	Prostate	-	3023 (100.0)	3023
4	Cervix Uteri	2230 (100.0)	-	2230
5	Non-Hodgkin Lymphoma	772 (44.7)	957 (55.3)	1729
6	Colorectum	777 (48.6)	821 (51.4)	1598
7	Stomach	586 (39.1)	912 (60.9)	1498
8	Soft Tissue	588 (48.8)	618 (51.2)	1206
9	Skin	442 (50.9)	427 (49.1)	869
10	Lung	343 (45.0)	419 (55.0)	762
11	Corpus Uteri	654 (100.0)	-	654
12	Brain/CNS	265 (50.9)	256 (49.1)	521
13	Hodgkin Lymphoma	193 (42.2)	264 (57.8)	457
14	Thyroid	376 (82.6)	79 (17.4)	455
15	Ovary	382 (100)	-	382
16	Oral	140 (41.7)	196 (58.3)	336
17	Liver	88 (26.3)	247 (73.7)	335
18	Urinary Bladder	93 (31.7)	200 (68.3)	293
19	Pancreas	149 (51.6)	140 (48.4)	289
20	Melanoma	138 (51.5)	130 (48.5)	268

### Pediatric cancers

**Fig. 3:**
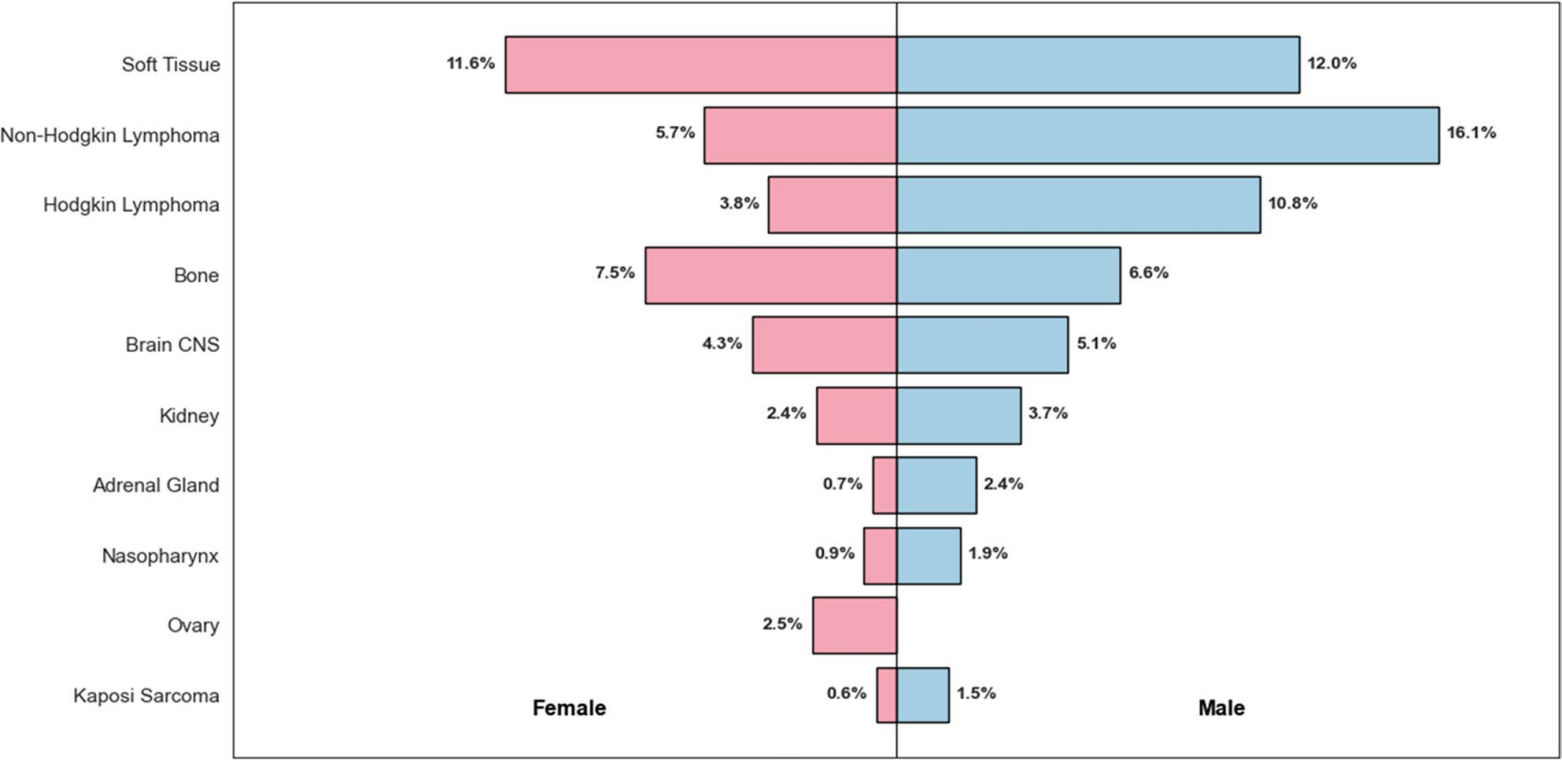
Sex distribution of top 10 pediatric cancer sites (2015–2024)

Pediatric cancer cases (≤ 19 years) accounted for 4.1% (1325/32,445) of all malignancies diagnosed during the study period. The leading malignancies in this age group were soft tissue tumors (199/1325; 19.4%), non-Hodgkin lymphoma (184/1325; 17.9%), and Hodgkin lymphoma (123/1325; 12.0%). A male predominance was observed in lymphomas and adrenal tumors (Table 3; Fig. 3).

**Table 3:** Summary of Top Pediatric Cancer Cases (Ages 0–19) by Cancer Site and Sex

Cancer Site	Female *n* (%)	Male *n* (%)	Overall *n* (%)
Soft Tissue	98 (11.6)	101 (12.0)	199 (19.4)
Non-Hodgkin Lymphoma	48 (5.7)	136 (16.1)	184 (17.9)
Hodgkin Lymphoma	32 (3.8)	91 (10.8)	123 (12.0)
Bone	63 (7.5)	56 (6.6)	119 (11.6)
Brain CNS	36 (4.3)	43 (5.1)	79 (7.7)
Kidney	20 (2.4)	31 (3.7)	51 (5.0)
Adrenal Gland	6 (0.7)	20 (2.4)	26 (2.5)
Nasopharynx	8 (0.9)	16 (1.9)	24 (2.3)
Ovary	21 (2.5)	0 (0.0)	21 (2.0)
Kaposi Sarcoma	5 (0.6)	13 (1.5)	18 (1.8)

### Regional sources of referral

**Fig. 4:**
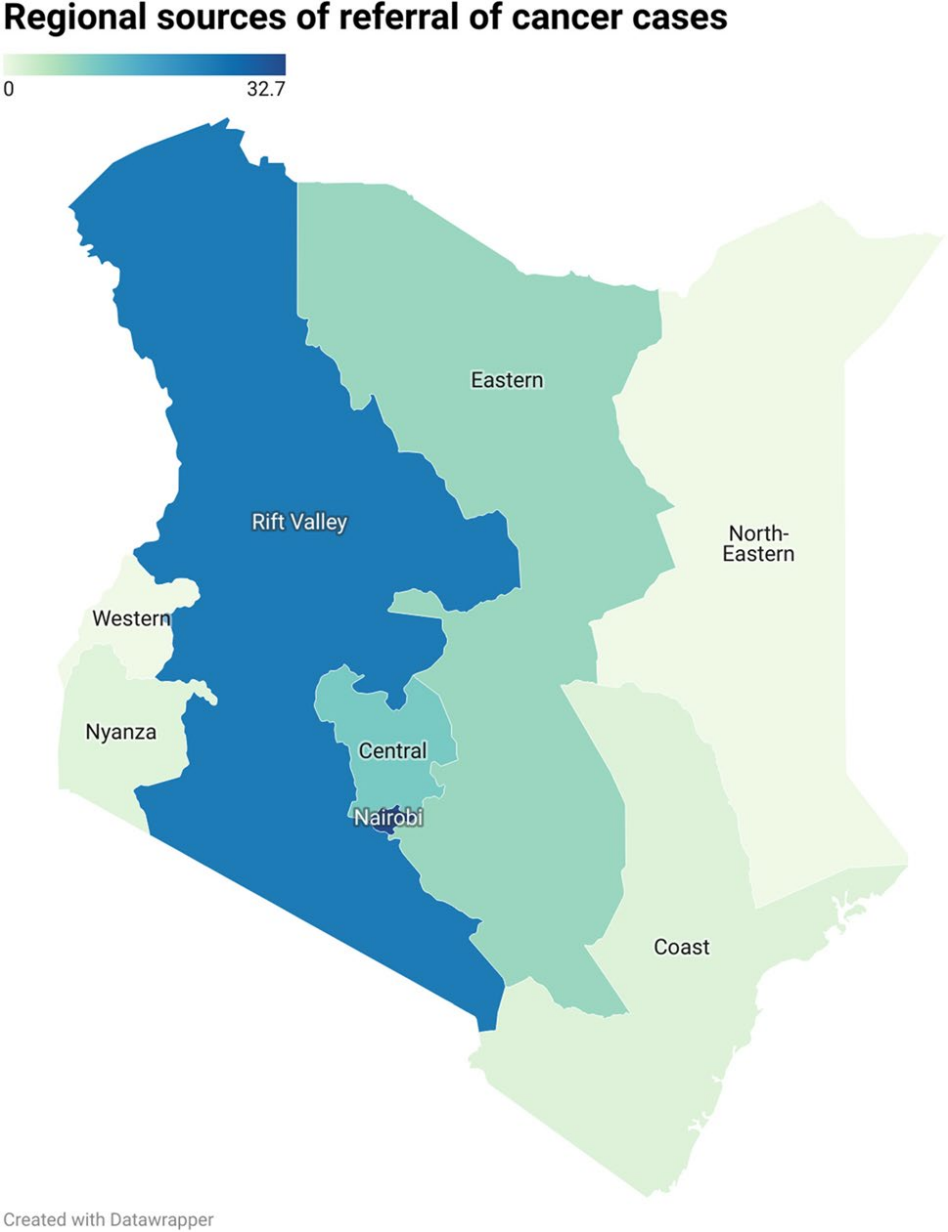
Map of Kenya showing regional sources of cancer referrals at AKUH, 2015–2024. Legend: Darker shading represents higher proportions of cases. The largest proportions were from Nairobi (32.7%) and Rift Valley (24.3%), while Western and North-Eastern regions contributed no cases

**Fig. 5:**
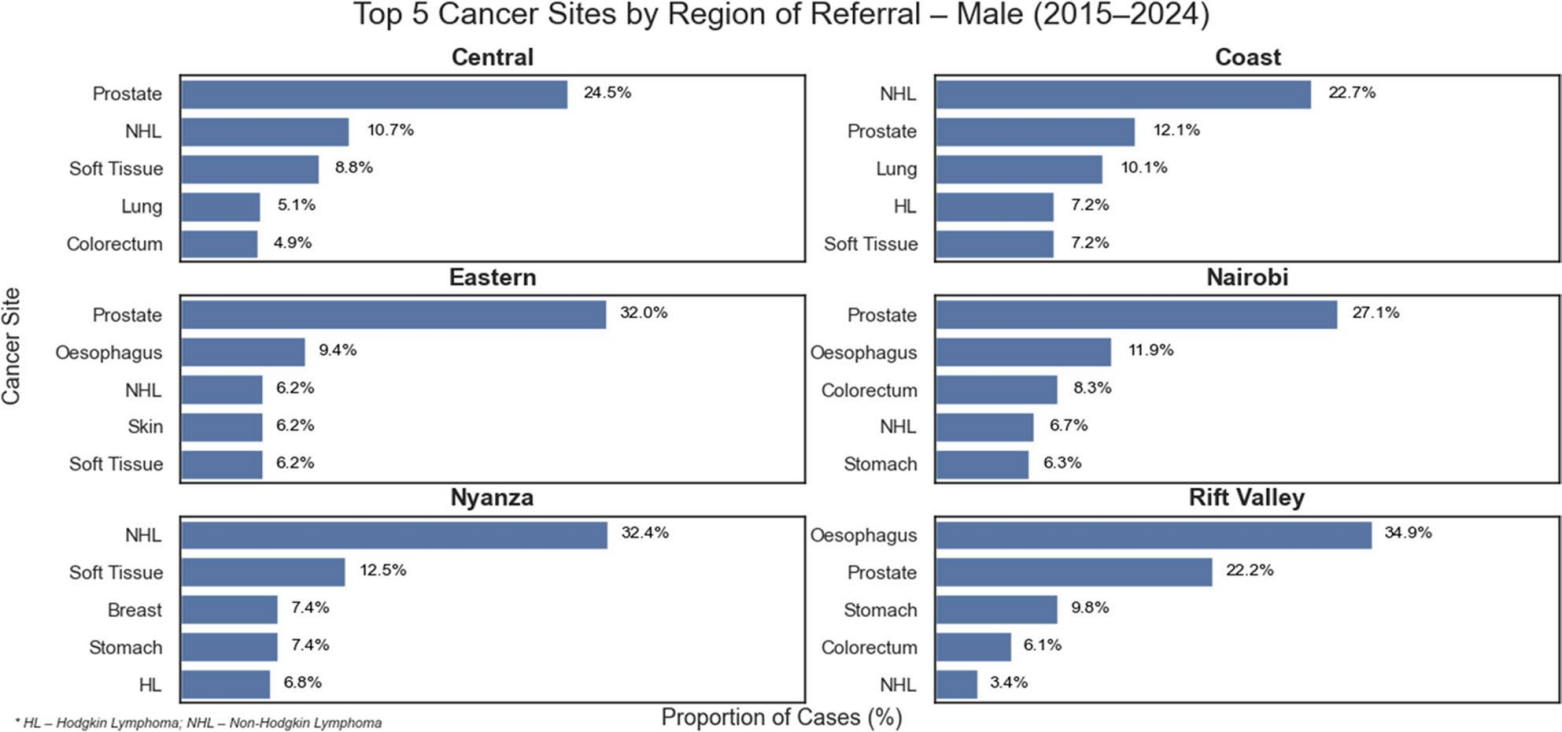
Illustrates the top five cancer sites in each region for males

**Fig. 6:**
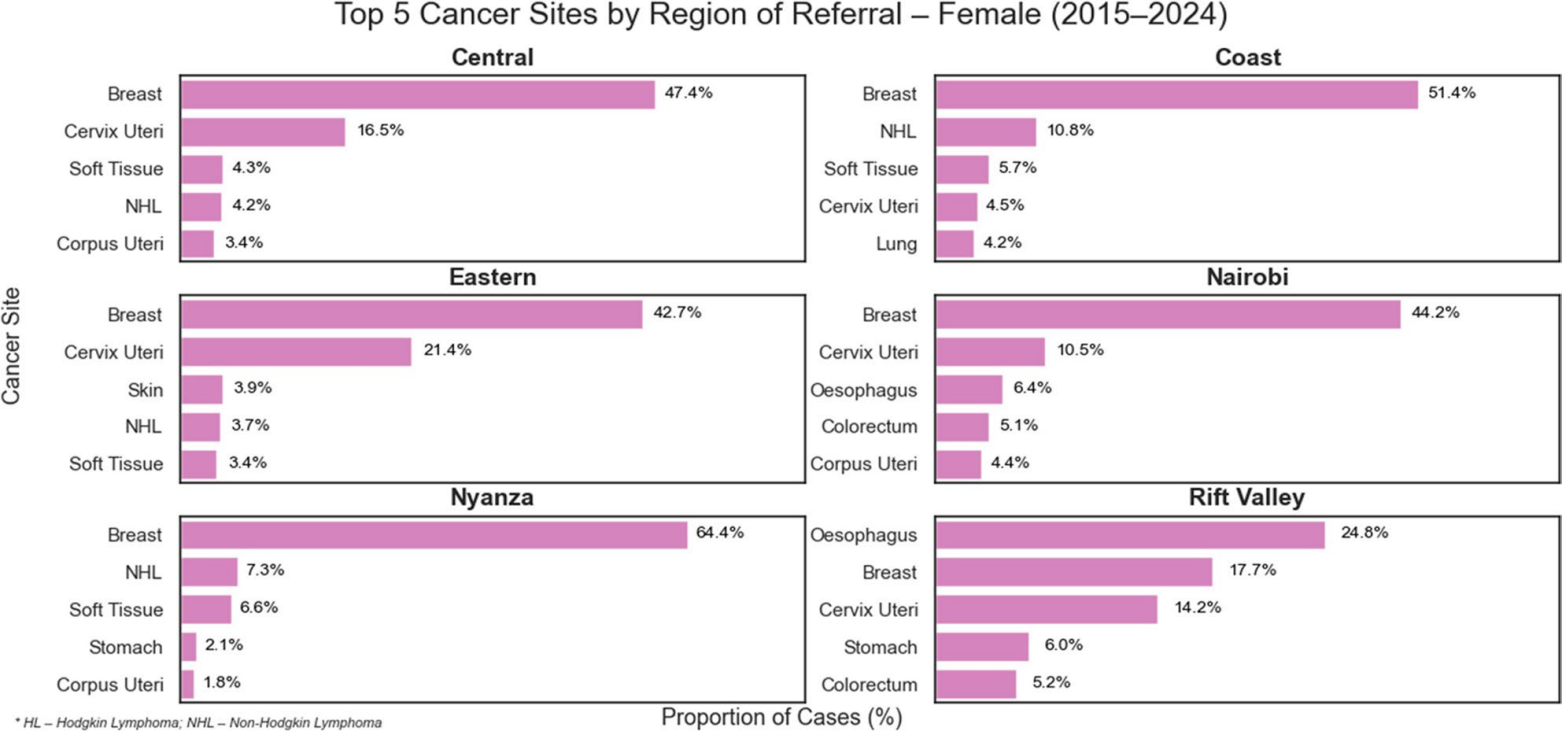
Illustrates the top five cancer sites in each region for females

The highest proportion of cases originated from Nairobi (32.7%) and Rift Valley (24.3%), with Central (10.6%) and Eastern (8.0%) contributing fewer cases. Coast (1.7%) and Nyanza (1.5%) had minimal referrals, while no cases were reported from Western or North-Eastern regions. Breast cancer dominated referrals from nearly all regions among women, while prostate and esophageal cancers varied by region among men. The Rift Valley contributed a disproportionate share of esophageal (2567/3839; 67%) and stomach (680/1498; 45%) cancer cases compared to other regions, as shown in Tables 4 and 5; Figs. 4, 5 and 6.

**Table 4:** Top 15 Cancer sites by Region (2015–2024)

Cancer Site	Nairobi	Rift Valley	Central	External, Unspecified	Eastern	Coast	Nyanza	International	Total
Breast	3022 (31.4%)	827 (10.9%)	1221 (38.7%)	945 (35.0%)	802 (33.5%)	177 (37.0%)	226 (50.3%)	16 (20.5%)	7236 (27.3%)
Esophagus	957 (9.9%)	2567 (33.9%)	71 (2.2%)	93 (3.4%)	133 (5.6%)	10 (2.1%)	5 (1.1%)	3 (3.8%)	3839 (14.5%)
Prostate	1222 (12.7%)	946 (12.5%)	279 (8.8%)	207 (7.7%)	326 (13.6%)	25 (5.2%)	11 (2.4%)	7 (9.0%)	3023 (11.4%)
Cervix Uteri	689 (7.2%)	616 (8.1%)	413 (13.1%)	115 (4.3%)	376 (15.7%)	15 (3.1%)	5 (1.1%)	1 (1.3%)	2230 (8.4%)
Non-Hodgkin Lymphoma	553 (5.7%)	269 (3.5%)	226 (7.2%)	374 (13.9%)	127 (5.3%)	83 (17.3%)	81 (18.0%)	16 (20.5%)	1729 (6.5%)
Colorectum	715 (7.4%)	486 (6.4%)	129 (4.1%)	134 (5.0%)	94 (3.9%)	23 (4.8%)	13 (2.9%)	4 (5.1%)	1598 (6.0%)
Stomach	469 (4.9%)	680 (9.0%)	94 (3.0%)	126 (4.7%)	84 (3.5%)	20 (4.2%)	20 (4.5%)	5 (6.4%)	1498 (5.7%)
Soft Tissue	380 (3.9%)	261 (3.4%)	207 (6.6%)	150 (5.6%)	122 (5.1%)	34 (7.1%)	44 (9.8%)	8 (10.3%)	1206 (4.6%)
Skin	287 (3.0%)	248 (3.3%)	120 (3.8%)	68 (2.5%)	132 (5.5%)	5 (1.0%)	3 (0.7%)	6 (7.7%)	869 (3.3%)
Lung	357 (3.7%)	46 (0.6%)	80 (2.5%)	216 (8.0%)	15 (0.6%)	35 (7.3%)	10 (2.2%)	3 (3.8%)	762 (2.9%)
Corpus Uteri	291 (3.0%)	151 (2.0%)	84 (2.7%)	53 (2.0%)	56 (2.3%)	10 (2.1%)	6 (1.3%)	3 (3.8%)	654 (2.5%)
Brain/CNS	211 (2.2%)	144 (1.9%)	73 (2.3%)	62 (2.3%)	18 (0.8%)	8 (1.7%)	3 (0.7%)	2 (2.6%)	521 (2.0%)
Hodgkin Lymphoma	166 (1.7%)	66 (0.9%)	63 (2.0%)	92 (3.4%)	35 (1.5%)	19 (4.0%)	16 (3.6%)	0 (0.0%)	457 (1.7%)
Thyroid	167 (1.7%)	186 (2.5%)	32 (1.0%)	25 (0.9%)	37 (1.5%)	4 (0.8%)	1 (0.2%)	3 (3.8%)	455 (1.7%)
Ovary	137 (1.4%)	88 (1.2%)	64 (2.0%)	38 (1.4%)	38 (1.6%)	11 (2.3%)	5 (1.1%)	1 (1.3%)	382 (1.4%)

**Table 5:** Ratio of esophageal cancers to colorectal cancer in nairobi compared with rift valley

Region	Esophageal cases	Colorectal cases	Ratio (Esophagus: Colorectum)
Nairobi	957	715	1.3:1
Rift Valley	2567	486	5.3:1

### Completeness of pathology reports

**Fig. 7:**
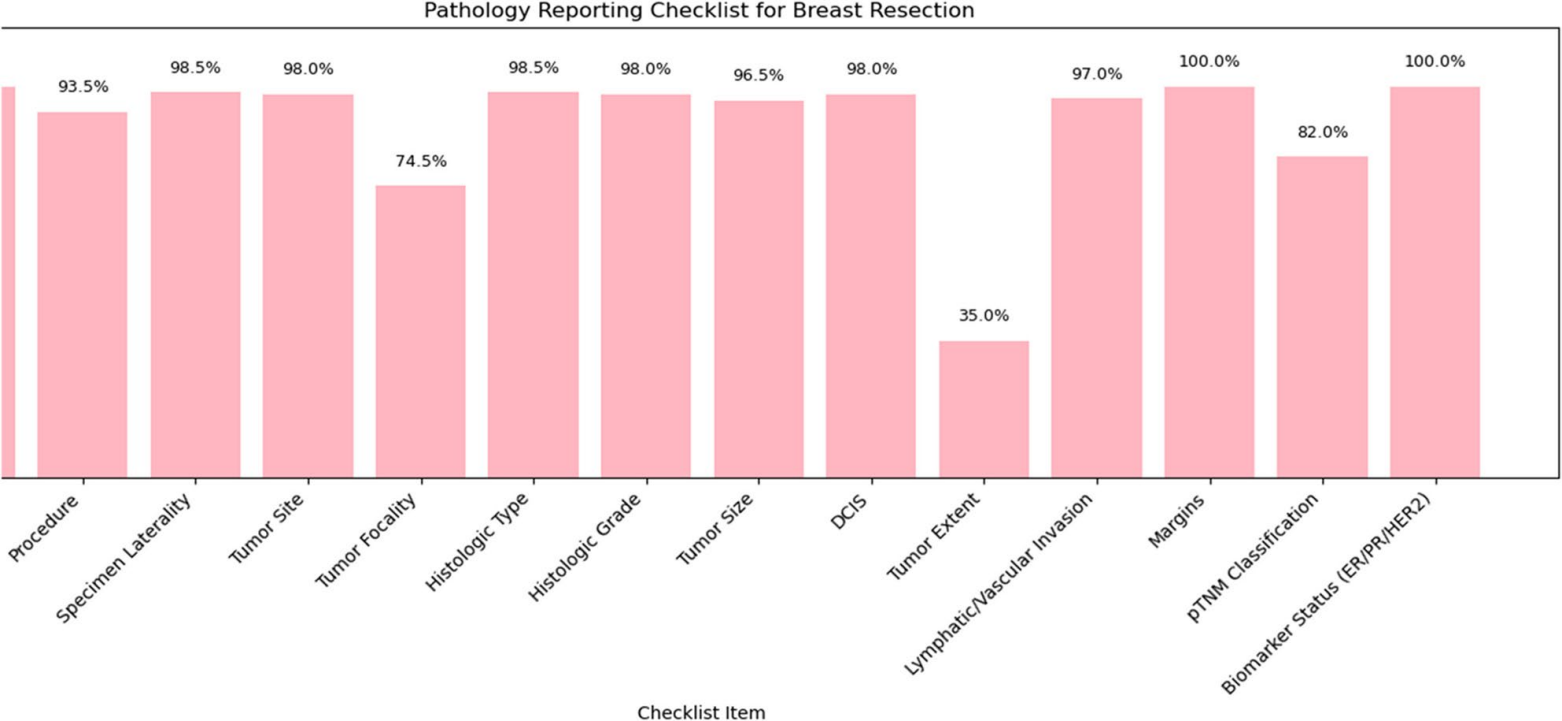
Shows the level of completeness in reporting breast surgical specimens as per CAP requirements

**Fig. 8:**
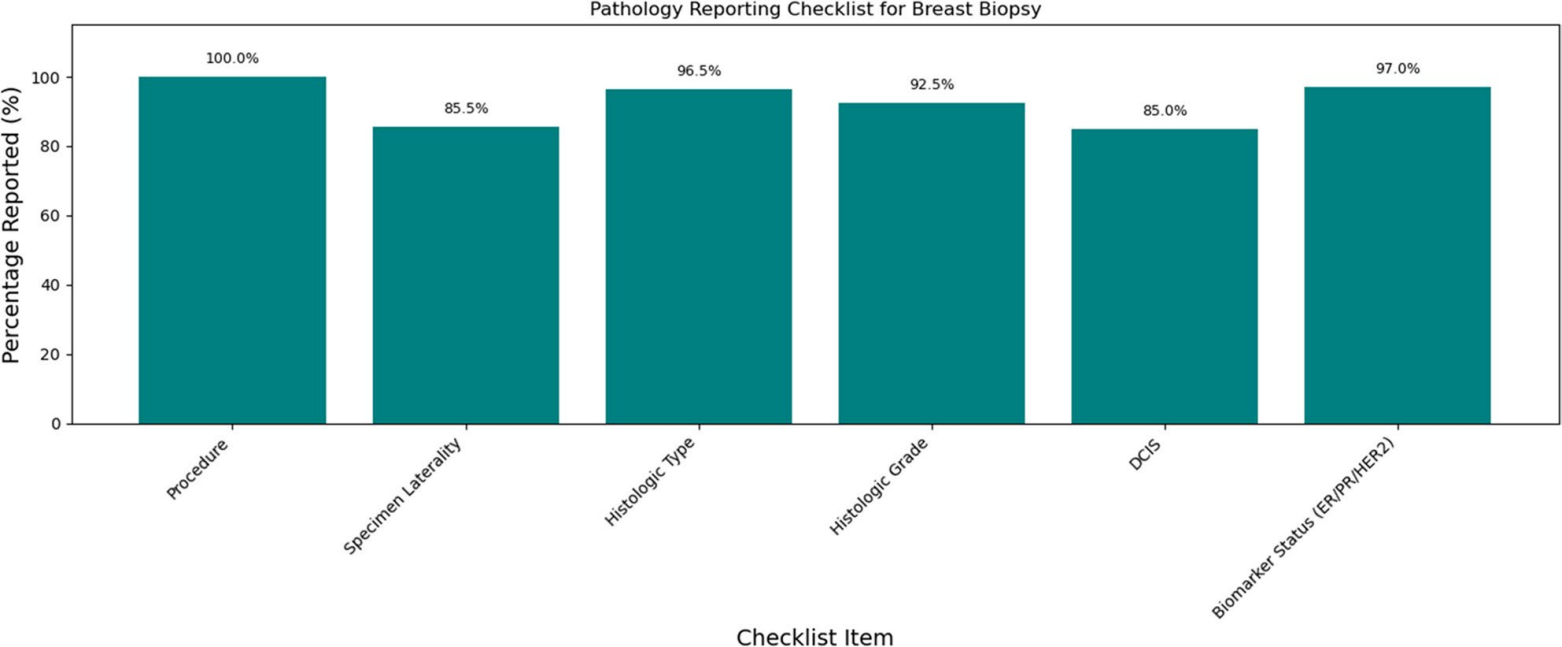
Completeness of pathology reporting for breast biopsy specimens

**Fig. 9:**
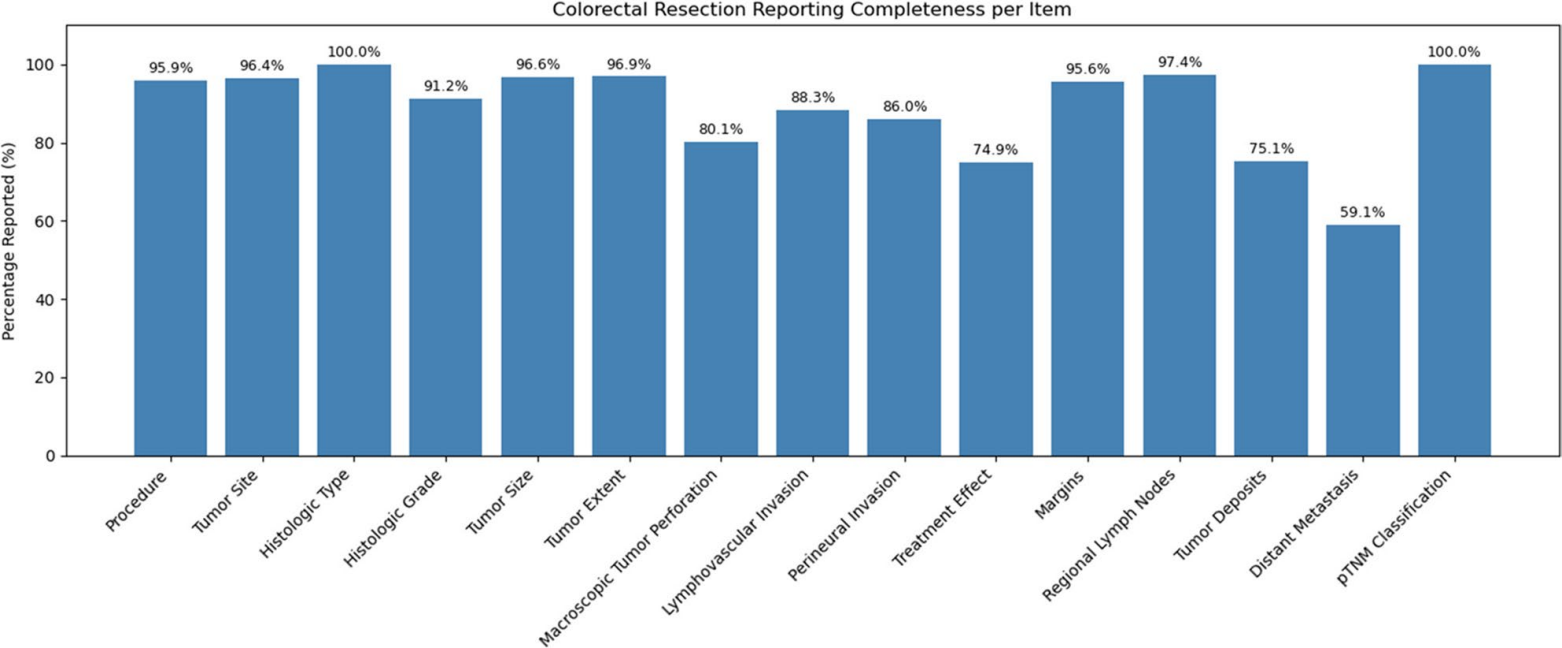
shows the completeness of pathology reporting for colorectal resection specimens

**Fig. 10:**
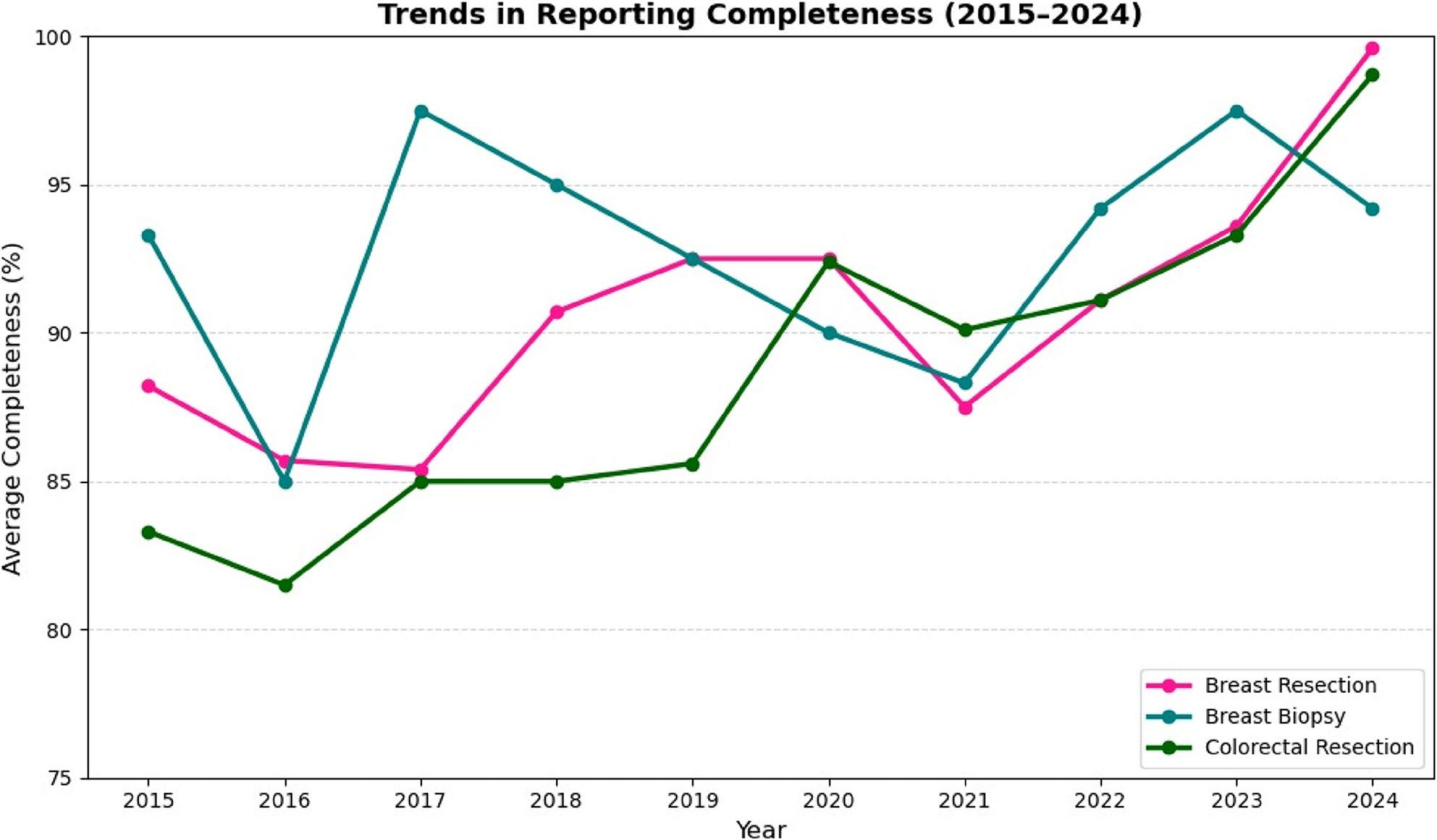
Shows the trend over the years of completeness in reporting breast and colorectal specimens

Completeness of cancer reporting generally improved over the decade. For breast cancer surgical specimens, most elements exceeded 95% completeness, though tumor extent (35.0%) and tumor focality (74.5%) were less frequently reported (Fig. 7). Breast biopsy reports consistently captured biomarkers (> 95%) but less frequently documented laterality and presence of DCIS (~ 85%) (Fig. 8). For colorectal cancer resections, key elements (histologic type, TNM) were reported in ≥ 95% of cases, but distant metastasis (59.1%) and treatment effect (74.9%) were underreported (Fig. 9). Overall completeness of cancer reporting improved from 83.3% (2015) to > 98% (2024; Fig. 10).

## Discussion

This 10-year pathology-based study at a tertiary referral center in Nairobi provides one of the largest pathology-based descriptions of histologically confirmed cancer diagnoses in Kenya to date. Over 32,000 cases were analyzed, with a female predominance and a younger median age of diagnosis in women than in men. Breast and prostate cancers were the most common cancers in females and males, respectively, consistent with national and global estimates. Pathology reporting for breast and colorectal cancers demonstrated high completeness in core CAP-recommended data elements, with improvements observed over time.

Based on the pattern of cancer cases diagnosed at our facility, international trends are reflected whereby cancer frequency rises after age 40, with women predominating, particularly at younger ages largely due to breast and cervical cancers [1]. Prostate cancer dominated among men, while breast and cervical cancers were the most frequently diagnosed cancers among women, findings that have also been documented elsewhere in Africa. In Nigeria, for instance, a hospital based registry study found that prostate cancer and breast cancer were the leading cancers diagnosed in males and females respectively [10]. These findings also compare well with GLOBOCAN estimates for the top cancers in Kenya, estimates that are derived from the two high-quality population-based registries in Kenya —Nairobi and Eldoret—covering approximately 11% of the population [1, 2]. A local study in rural Kenya (Nyandarua County) further supports this age and sex distribution. The researchers in Nyandarua aimed to establish a population-based cancer registry by collecting data from cancer patients in multiple institutions in the county. They reported a female predominance (54.9%), and earlier age at diagnosis among women compared to men. In both studies, women were most affected in the middle decades (41–60 years), while men presented at older ages [11].

For this study, pediatric cancers were defined as malignancies occurring in individuals aged 0–19 years (children and adolescents), consistent with the ICCC-3 classification [12]. Children and adolescents in our study accounted for 4.1% of diagnoses, in line with low global estimates. In a large population-based analysis of 153 cancer registries across 62 countries, Steliarova-Foucher et al. reported an age-standardized incidence rate (ASR) of 140.6 per million person-years among children aged 0–14 years and 185.3 per million among those aged 15–19 years. In contrast, global estimates from Bray et al. indicate that the overall cancer incidence rate for all ages combined was 212.5 per 100,000 (2125 per million) in men and 186.2 per 100,000 (1862 per million) in women in 2022. These figures demonstrate that malignancies are much more common in adults than in children. Other supporting estimates suggest childhood cancers represent only 1–2% of all cancer cases worldwide [13]. Locally Kamita and others found an incidence of 1.2% for pediatric cancer cases based on a population-based registry study in Nyandarua county of Kenya [11]. Lymphomas and soft tissue tumors were the leading cancers, and male predominance was also observed, reflecting established regional and global patterns [14].

Referral data showed that most cancers originated from Nairobi and the Rift Valley, with notable geographic variation by site. Esophageal and stomach cancers were disproportionately referred from the Rift Valley. This is demonstrated in our data by the high ratio of esophageal carcinoma to colorectal carcinoma referred from Rift valley (5.3:1 compared to a ratio of 1.3:1 in Nairobi). This implies a higher proportion of esophageal carcinoma cases are referred from the Rift valley province compared to other regions. It echoes previous studies linking regional practices such as consumption of hot beverages, mursik (a traditional fermented milk beverage), and home-brewed alcohol, among others, to esophageal cancer risk in the Rift valley region of Kenya [15, 16]. By contrast, breast and prostate cancers were more frequently referred from Nairobi, which is the capital and largest city, suggesting higher awareness and access to screening and diagnostic services in urban areas. Sparse referrals from far-flung and rural areas like Nyanza, Coast, Western and Northeastern regions raise concern for under-diagnosis or under-referral, underscoring the need for decentralized diagnostic capacity and equitable screening programs.

The high levels of completeness for key pathology data elements found in this study, including surgical margins, histologic type, biomarkers, and pTNM classification demonstrate compliance with structured reporting. Improvements over time likely reflect adoption of standardized templates. Another factor is the increasing role of multidisciplinary tumor boards, consistent with international evidence that these enhance report quality [8, 9]. The laboratory has been CAP accredited since the year 2018, and this may have also contributed to the stricter compliance with structured reporting.

### Strengths and limitations

The main strength of this study is the large dataset spanning a decade, with histologically confirmed cancer diagnoses, providing valuable information on trends over time. Limitations include the reliance of this registry on cases retrieved from the AKUH pathology department database only. As a private tertiary hospital, a proportion of patients receiving direct care at AKUH represent middle- and higher-income groups. This potential socioeconomic bias is mitigated, but not eliminated, by the fact that the AKUH laboratory also receives substantial referrals from low-cost public hospitals and is one of the service providers in the national social health insurance scheme. There is also underrepresentation of cancers that can be diagnosed clinically or radiologically without the need for pathology such as hepatocellular carcinomas. There is lack of complete staging, treatment and survival data for these pathology-based cases limiting utilization of this information. Another limitation is that the absence of a defined population denominator makes it impossible to calculate reliable incidence rates. Additionally, we were unable to compare pathology-based diagnoses with clinical treatment registries, which may further characterize discordance between diagnostic and treatment patterns. Nonetheless, these data provide critical insights into cancer distribution in Kenya and highlight the role of private sector pathology services in bridging diagnostic gaps.

### Conclusion and recommendations

This ten-year pathology-based review of 32,445 cancer cases provides important insights into the epidemiology of cancer in Kenya. Breast and prostate cancers were the leading malignancies diagnosed among females and males, respectively, with most cases diagnosed between ages 41–70 years. Regional variations were evident, notably the high proportion of esophageal and stomach cancers referred from the Rift Valley. The overall completeness of pathology reporting for major cancers, including breast and colorectal, was high, reflecting adherence to international standards.

To strengthen cancer control, pathology-based registries should be integrated into the national surveillance framework to complement population-based systems. Addressing regional disparities through targeted prevention, and improving diagnostic capacity in underserved regions such as Nyanza, Western and North-Eastern Kenya are priorities. Continued professional training and regular audits will enhance reporting consistency, while public–private partnerships can expand equitable access to diagnostic services nationwide.

## Supplementary Information

Supplementary Material 1.

## Data Availability

The datasets used and/or analyzed during the current study are available from the corresponding author on reasonable request.

## References

[1] Bray F, Laversanne M, Sung H, Ferlay J, Siegel RL, Soerjomataram I, et al. Global cancer statistics 2022: GLOBOCAN estimates of incidence and mortality worldwide for 36 cancers in 185 countries. CA Cancer J Clin. 2024;74(3):229–63. [cited 2025 Aug 23]. 10.3322/caac.21834. 38572751 10.3322/caac.21834

[2] Ministry of Health. The National Cancer Control Strategy. (2023–2027). 2023. [cited 2025 Aug 23]. Available from: http://guidelines.health.go.ke:8000/media/NATIONAL_CANCER_CONTROL_STRATEGY_2023-2027_7uTQQP4.pdf.

[3] Bray F, Znaor A, Cueva P, Korir A, Swaminathan R, Ullrich A et al. The role and status of population-based cancer registration. 2014. [cited 2025 Aug 24]; Available from: https://www.ncbi.nlm.nih.gov/books/NBK566966/. 33502836

[4] Korir A, Gakunga R, Subramanian S, Okerosi N, Chesumbai G, Edwards P, et al. Economic analysis of the Nairobi Cancer Registry: Implications for expanding and enhancing cancer registration in Kenya. Cancer Epidemiol. 2016;45(Suppl 1):520–9. [cited 2025 Aug 23]. Available from: https://pubmed.ncbi.nlm.nih.gov/27915004/. 10.1016/j.canep.2016.11.006 PMC5840871 27915004

[5] Jedy-Agba EE, Curado MP, Oga E, Samaila MO, Ezeome ER, Obiorah C, et al. The role of hospital-based cancer registries in low and middle income countries-The Nigerian Case Study. Cancer Epidemiol. 2012;36(5):430–5. [cited 2025 Aug 23]. Available from: https://pubmed.ncbi.nlm.nih.gov/22704971/. 22704971 10.1016/j.canep.2012.05.010 PMC3438360

[6] Bhurgri Y, Hasan SH, Pervez S, Kayani N, Hussainy AS, Muzaffar S et al. Large-scale pathology-based cancer data - A reflection of population-based cancer data. Pathol Oncol Res. 2002;8(1):62–7. [cited 2025 Aug 23]. Available from: https://link.springer.com/article/10.1007/BF03033704. 10.1007/BF03033704 11994766

[7] Brand NR, Wolf N, Flanigan J, Njoroge R, Karagu A. Histology and cytopathology capacity in the public health sector in Kenya. J Glob Oncol. 2018;2018(4):1–7. [cited 2025 Aug 23]. Available from: https://pubmed.ncbi.nlm.nih.gov/30241145/. 10.1200/JGO.17.00122 PMC6180785 30241145

[8] Ebben KCWJ, Sieswerda MS, Luiten EJT, Heijns JB, van der Pol CC, Bessems M, et al Impact on Quality of Documentation and Workload of the Introduction of a National Information Standard for Tumor Board Reporting. JCO Clin Cancer Inf. 2020;4(4):346–56. [cited 2025 Aug 23]. Available from: https://pubmed.ncbi.nlm.nih.gov/32324446/. 10.1200/CCI.19.00050 PMC7444641 32324446

[9] Schaad N, Berezowska S, Perren A, Hewer E Impact of template-based synoptic reporting on completeness of surgical pathology reports. Virchows Arch. 2024;484(1):31–6. [cited 2025 Aug 23]. Available from: https://link.springer.com/article/10.1007/s00428-023-03533-6. 37017774 10.1007/s00428-023-03533-6 PMC10791929

[10] Yusuf I, Atanda AT, Umar AB, Imam MI, Mohammed AZ, Ochicha O, et al. Cancer in Kano, Northwestern Nigeria: A 10–year Update of the Kano Cancer Registry. Ann Trop Pathol. 2017;8(2):87–93. [cited 2025 Oct 30]. Available from: https://www.ajol.info/index.php/atp/article/view/274618.

[11] Kamita M, Waweru H, Githinji M, Kibiro E, Makokha F. Establishing a cancer registry and baseline data for Nyandarua County, Kenya: A step towards establishing a central cancer registry. Heliyon. 2024;10(20). [cited 2025 Aug 24]. Available from: https://www.cell.com/action/showFullText?pii=S2405844024150876. 10.1016/j.heliyon.2024.e39056 PMC11530777 39492911

[12] Steliarova-Foucher E, Stiller C, Lacour B, Kaatsch P. International classification of childhood cancer, third edition. Cancer. 2005;103(7):1457–67. [cited 2025 Aug 24]. Available from: https://pubmed.ncbi.nlm.nih.gov/15712273/. 15712273 10.1002/cncr.20910

[13] Pineros M, Mery L, Soerjomataram I, Bray F, Steliarova-Foucher E. Scaling Up the Surveillance of Childhood Cancer: A Global Roadmap. JNCI J Natl Cancer Inst. 2020;113(1):9. [cited 2025 Aug 24]. Available from:. https://pmc.ncbi.nlm.nih.gov/articles/PMC7781445/. 10.1093/jnci/djaa069 PMC7781445 32433739

[14] Stefan DC. Childhood cancer in Africa: An overview of resources. J Pediatr Hematol Oncol. 2015;37(2):104–8. [cited 2025 Aug 23]. Available from: https://pubmed.ncbi.nlm.nih.gov/24487917/. 24487917 10.1097/MPH.0000000000000111

[15] Middleton DRS, Menya D, Kigen N, Oduor M, Maina SK, Some F, et al. Hot beverages and oesophageal cancer risk in western Kenya: Findings from the ESCCAPE case–control study. Int J Cancer. 2019;144(11):2669. [cited 2025 Aug 23]. Available from: https://pmc.ncbi.nlm.nih.gov/articles/PMC6519248/. 30496610 10.1002/ijc.32032 PMC6519248

[16] Patel K, Wakhisi J, Mining S, Mwangi A, Patel R. Esophageal Cancer, the Topmost Cancer at MTRH in the Rift Valley, Kenya, and Its Potential Risk Factors. ISRN Oncol. 2013;2013:503249. [cited 2025 Aug 23]. Available from: https://pmc.ncbi.nlm.nih.gov/articles/PMC3893746/. 24490085 10.1155/2013/503249 PMC3893746

