# Supplementary material for: Ten-year pathology-based cancer registry at a tertiary referral hospital in Kenya (2015–2024): distribution of cancers and completeness of pathology reporting
Source: BMC Cancer. 2026 Mar 9;26:485. doi: 10.1186/s12885-026-15813-w (PMC13085426; doi:10.1186/s12885-026-15813-w)
Supplement: Supplementary file 1 — Supplementary Material 1. [file 12885_2026_15813_MOESM1_ESM.docx]

| **Cancer Site** | **Female**  **n (%)** | **Male**  **n (%)** | **Overall** |
| --- | --- | --- | --- |
| BREAST | 6926 (95.7) | 310 (4.3) | 7236 |
| OESOPHAGUS | 1610 (41.9) | 2229 (58.1) | 3839 |
| PROSTATE | 0 (0.0) | 3023 (100.0) | 3023 |
| CERVIX UTERI | 2230 (100.0) | 0 (0.0) | 2230 |
| CANCER OF UNKNOWN PRIMARY | 985 (54.1) | 837 (45.9) | 1822 |
| NON-HODGKIN LYMPHOMA | 772 (44.7) | 957 (55.3) | 1729 |
| COLORECTUM | 777 (48.6) | 821 (51.4) | 1598 |
| STOMACH | 586 (39.1) | 912 (60.9) | 1498 |
| SOFT TISSUE | 588 (48.8) | 618 (51.2) | 1206 |
| SKIN | 442 (50.9) | 427 (49.1) | 869 |
| LUNG | 343 (45.0) | 419 (55.0) | 762 |
| CORPUS UTERI | 654 (100.0) | 0 (0.0) | 654 |
| BRAIN CNS | 265 (50.9) | 256 (49.1) | 521 |
| HODGKIN LYMPHOMA | 193 (42.2) | 264 (57.8) | 457 |
| THYROID | 376 (82.6) | 79 (17.4) | 455 |
| OVARY | 382 (100.0) | 0 (0.0) | 382 |
| LIP, ORAL CAVITY | 154 (42.2) | 211 (57.8) | 365 |
| LIVER | 88 (26.3) | 247 (73.7) | 335 |
| URINARY BLADDER | 93 (31.7) | 200 (68.3) | 293 |
| PANCREAS | 149 (51.6) | 140 (48.4) | 289 |
| MELANOMA | 138 (51.5) | 130 (48.5) | 268 |
| BONE | 124 (47.1) | 139 (52.9) | 263 |
| KAPOSI SARCOMA | 78 (30.2) | 180 (69.8) | 258 |
| NASOPHARYNX | 83 (31.7) | 179 (68.3) | 262 |
| KIDNEY | 104 (43.7) | 134 (56.3) | 238 |
| LARYNX | 38 (21.3) | 140 (78.7) | 178 |
| OTHER AND UNSPECIFIED MALIGNANT NEOPLASMS | 86 (50.0) | 86 (50.0) | 172 |
| SMALL INTESTINE | 56 (41.5) | 79 (58.5) | 135 |
| SALIVARY GLAND | 66 (50.0) | 66 (50.0) | 132 |
| ANAL | 54 (43.5) | 70 (56.5) | 124 |
| EYE | 65 (53.3) | 57 (46.7) | 122 |
| VULVA | 93 (100.0) | 0 (0.0) | 93 |
| GALL BLADDER | 67 (72.8) | 25 (27.2) | 92 |
| NASAL AND PARANASAL | 43 (47.3) | 48 (52.7) | 91 |
| PLASMA CELL NEOPLASM | 30 (40.0) | 45 (60.0) | 75 |
| VAGINA | 72 (100.0) | 0 (0.0) | 72 |
| TESTIS | 0 (0) | 62 (100) | 62 |
| HYPOPHARYNX | 23 (42.6) | 31 (57.4) | 54 |
| ADRENAL GLAND | 16 (37.2) | 27 (62.8) | 43 |
| THYMUS | 15 (46.9) | 17 (53.1) | 32 |
| OROPHARYNX | 14 (39.3) | 21 (60.7) | 35 |
| OTHER BILIARY TRACT | 8 (50.0) | 8 (50.0) | 16 |
| APPENDIX | 7 (50.0) | 7 (50.0) | 14 |
| URETHRA | 9 (69.2) | 4 (30.8) | 13 |
| PENIS | 0 (0.0) | 13 (100.0) | 13 |
| PERITONEUM, OMENTUM AND MESENTERY | 6 (60.0) | 4 (40.0) | 10 |
| PLEURA | 2 (28.6) | 5 (71.4) | 7 |
| URETER | 3 (75.0) | 1 (25.0) | 4 |
| FALLOPIAN TUBE | 4 (100.0) | 0 (0.0) | 4 |
|  |  |  | 32,445 |
